# The interaction between gut microbiota and age-related changes in immune function and inflammation

**DOI:** 10.1186/1742-4933-10-31

**Published:** 2013-08-05

**Authors:** Thea Magrone, Emilio Jirillo

**Affiliations:** 1 Department of Basic Medical Sciences, Neuroscience and Sensory Organs, University of Bari, Policlinico, Piazza G. Cesare 11, 70124, Bari, Italy

**Keywords:** Ageing, Gut, Immunity, Microbiota

## Abstract

Intestinal microbiota and gut immune systems interact each other, maintaining a condition of homeostasis in the context of the intestinal habitat. However, both systems undergo modifications in elderly, thus accounting for a low grade inflammatory status which, in turn, may evolve toward more severe pathological conditions such as inflammatory bowel disease and colon rectal cancer. In addition, in western societies dietary habits may negatively influence the microbiota composition, also altering gut immune response which is *per se* impaired in elderly. In order to prevent the outcome of aged-related disease, supplementation of nutraceuticals able to correct abnormalities of both immune system and microbiota has become more frequent than in the past. In this respect, a better identification of components of the aged microbiota as well as a deeper analysis of gut mucosal immunity function should be pursued.

## Introduction

The intestinal microbiota is mostly confined in the colon where resides 1.5 Kg of microbes that is equal to about 10^14^ microorganims [1]. Human microbiota represents a “superorganism” possessing more genes than the human genome [2]. It undergoes individual variations in its composition and, in the same individual, variations in the different segments of the bowel have been reported [3]. Moreover, the microbiota of the mucosa seems to differ from that of the lumen and not always a direct interaction between microbiota and epithelial cells does occur [4]. Actually, two major *phyla* have been identified in the animal and human microbiota, such as Bacteroidetes (Gram-negative bacteria) and Firmicutes (Gram-positive bacteria). However, Actinobacteria and Protobacteria can predominantly colonize the intestine in some people [5,6].

In the context of the gut associated lymphoid tissue (GALT), enterocytes or intestinal epithelial cells (IECs) represent the first barrier against invading microorganisms either secreting mucin or defensins (a class of antimicrobial peptides) or sensing pathogens *via* Toll-like receptors (TLRs) [7]. Furthermore, microfolding (M) cells, specialized IEC, are able to sample microbial antigens and transfer them to lamina propria (LP) immune cells [*e.g.*, dendritic cells (DCs)] [8]. In turn, DCs act as presenting antigen cells (APCs), thus triggering both harmful and protective responses in the host [9]. DCs in the presence of a milieu enriched in interleukin (IL)-6, IL-1β and transforming growth factor (TGF)-β are able to polarize the immune response towards T helper (h)17 cells which, in turn, release IL-17A, IL-17 F, IL-21 and IL-22, thus becoming inflammatory in the presence of IL-23 [10]. This immune pathway is mainly activated in the course of inflammatory bowel disease (IBD).

On the other hand, CD103+ cells are tolerogenic and in the presence of IL-10, TGF-β, thymic stromal lymphopoietin and vasoactive intestinal peptide induce T regulatory (Treg) cells [11]. These CD4 + CD25 + FoxP3+ cells release IL-10 in the bowel, counteracting the activity of Th17 cells [12]. This tolerogenic anti-inflammatory activity is favored by retinoic acid (RA), a metabolite of vitamin A, produced by CD103+ tolerogenic DCs [13]. Of note, RA seems to directly interfere with Th17 polarization. In the context of intestinal mucosa, secretory (s) IgA production by B cells prevents bacterial adhesion to mucosal surfaces and neutralize toxins.

**Figure 1:**
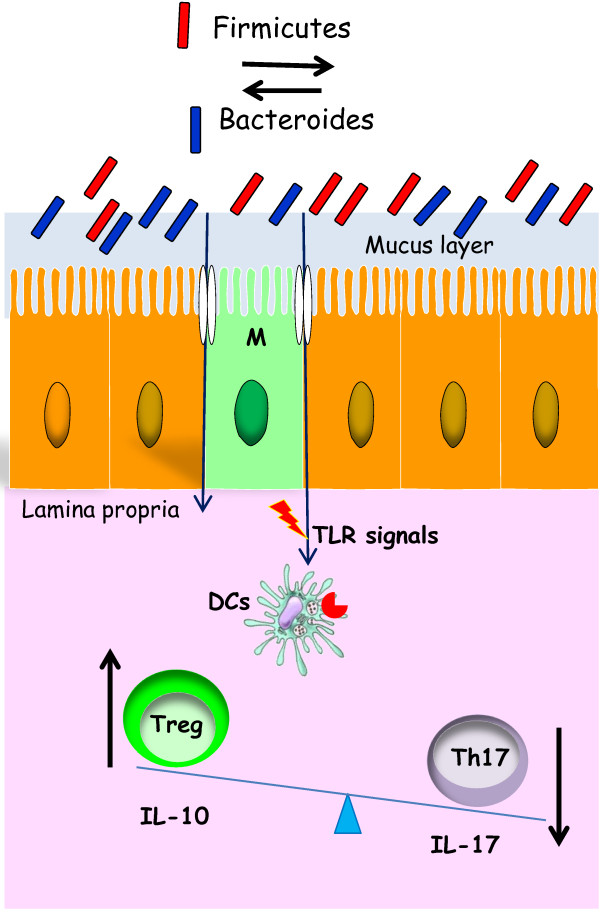
**Immune homeostatis maintained by the intestinal microbiota.** The equilibrium between Bacteroidetes and Firmicutes leads to the activation of T regulatory cells with production of the anti-inflammatory cytokine IL-10. On the other hand, release of IL-17 by Th17 cells is reduced.

Regulation of the intestinal immune homeostasis by the microbiota is illustrated in Figure 1.

In aged GALT, a marked multiple impairment of the immune response has been reported as evidenced by several studies conducted in animal models [14]. Major alterations are represented by [15]:

1. Reduced secretion of mucus and α-defensin;

2. Easy entry of pathogens into the mucosal layers and generation of a low grade inflammatory response (the so-called “inflamm-ageing”) [16] with Th1, Th2 and Th17 cell polarization.

This condition of inflamm-ageing [16] is perpetuated by overgrowth of intestinal pathobionts.

## Interactions between intestinal microbiota and immune system

**Figure 2:**
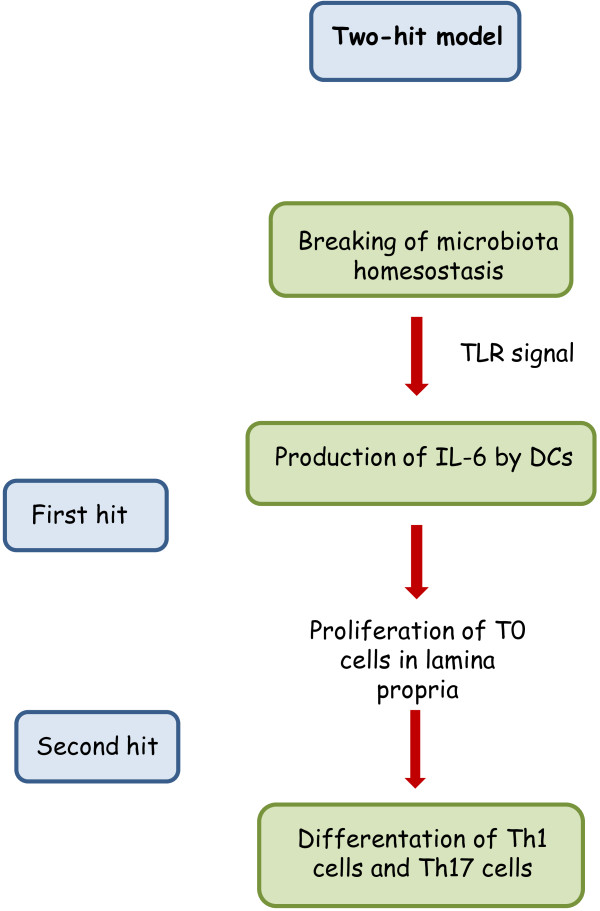
**The two-hit model in experimental colitis.** Alteration of the microbiota leads to the activation of DCs which produce IL-6 (first hit). In turn, IL-6 activates T0 cells which differentiate into Th1 cells and Th17 cells, respectively. This polarization of the immune response generates production of inflammatory cytokines (second hit).

Microbiota and immune cells actively interact within the gut [17]. Evidence has been provided that *Bacteroides fragilis* induces production of IL-10 by Treg cells *via* recognition of the polysaccharide A by TLR-2 [18]. In addition, lactobacilli and bifidobacteria play a tolerogenic role, rendering DCs less undifferentiated [19]. Conversely, segmented filamentous bacteria (SFB), component of the animal microbiota, are able to induce production of IL-17 from Th17 cells in mice [20]. Therefore, a fine balance is required in the daily interplay between microbiota and innate and adaptive immune cells to avoid noxious reactions to the host. According to the two-hit model [21] alteration of the microbiota triggers IL-6 production by lamina propria DCs, thus leading to activation of T0 cells. Differentiation of T0 cells into Th1 cells and Th17 cells creates an inflammatory milieu which culminates in colitis (Figure 2).

Studies on the aged intestinal microbiota have led to conflicting results. A decline of bifidobacteria and lactobacilli has been reported in the elderly with an increase of Bacteroides and facultative anaerobes [22,23]. In contrast, others reported higher levels of Ruminococcus and lower levels of Eubacterium and Bacteroides [24] with higher levels of bifidobacteria in comparison with the younger counterpart [25]. Finally, no differences between aged and younger individuals have been reported by others except for higher numbers of aerobes in elderly [26]. Also differences in aged microbiota were found depending on the country examined. In this respect, in a small population of aged Italian subjects an unchanged level of Bacteroidetes and an increase in *Faecalibacterium* spp. were observed [27]. *Viceversa* in a large cohort of Irish elderly people Bacteroidetes and *Faecalibacterium* spp. remarkably increased [22]. In the above mentioned group of Italian people no differences in microbiota were found when young adults (30 yrs old) and elderly (70 yrs old) were compared. Conversely, in the same group, centenarians exhibited a different composition of their microbiota. While Bacteroidetes and Firmicutes were still present with levels comparable to those of younger adults, a decrease of Clostridium cluster XIVa, an increase in bacilli and rearrangement of Clostridium cluster IV were reported [27]. In addition, in centenarians the observed increase in Proteobacteria, the so-called “pathobionts”, may explain the high frequency of infections once these bacteria have escaped from the host immune response [28].

**Figure 3:**
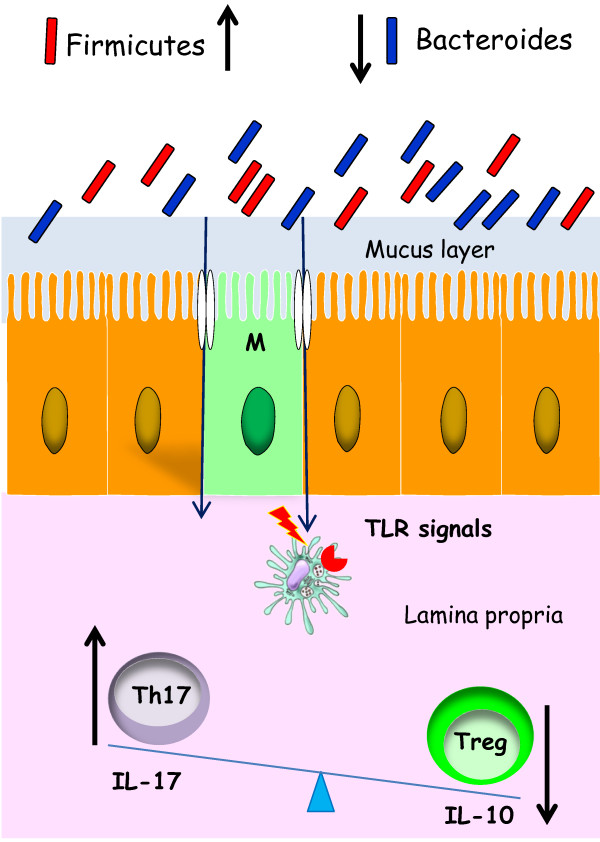
**Effects of altered microbiota in the intestinal immune response in elderly people.** Even if data on the aged microbiota are still controversial, in some cases increase of Firmicutes and decrease of Bacteroidetes may lead to a switch of the immune response towards an inflammatory profile with activation of Th17 cells.

Microbiota components account for the production of short chain fatty acids (SCFA) and, in particular butyrate, acetate and propionate. SCFA are endowed with anti-inflammatory (inhibition of NF-κB) and anti-neoplastic activities, also exerting a protective function in favor of intestinal epithelia [29]. In fact, butyrate has been shown to provide energy to the intestinal epithelium, as suggested by epithelial atrophy and inflammation in diversion colitis owing to SCFA deficiency [30]. In aged people, evidence has been provided that reduction of butyrate levels is depending on the decreased number of *Faecalibacterium* (F.) *prausnitzii*, *Eubacterium hallii* and *Eubacterium rectal*/Roseburia group [27]. Therefore, SCFA decrease may lead to an impaired secretion of mucins by the IECs and, therefore, easier entry of pathogens into the intestinal mucosa, especially Enterobacteriaceae. These Gram-negative bacteria are able to release lipopolysaccharides or endotoxins, which, in turn, aggravate the inflammatory condition [31]. In general terms, patients with IBD exhibit an abnormal microbiota with instability of dominant species which is higher than in healthy controls. In particular, *F. Prausnitzii* is severely reduced in Chron’s disease and in ulcerative colitis with an increased prevalence of adherent-invasive *E. coli* strains. However, the question is still open whether this alteration of microbiota is the cause or the consequence of IBD [32]. Moreover, evidence has been provided for a decreased content of SCFA in colon rectal cancer (CRC) with an increase of CRC in the western elderly population. A condition of chronic inflammation dependent on the change of microbiota leading to TLR-mediated NF-κB activation and colonization of the bowel by toxigenic bacterial strains, such as *Helicobacter pylori*, *Bacteroides fragilis* and *Escherichia coli* seems to contribute to the pathogenesis of CRC [33]. In this framework, in a recent study a comparison of aged microbiota was made between community-dwelling individuals and long-stay individuals. Actually, SCFA fecal content was more pronounced in community group than in long-stay patients [34]. In the latter, IL-6, IL-8 and C-reactive protein levels were higher than in the former group, as expression of a status of systemic inflammation. All these evidences correlated to a change in microbiota since in community individuals a higher numbers of Firmicutes and lower numbers of Bacteroidetes than those observed in long-stay patients were detected [34]. This situation is depicted in Figure 3 where the activation of Th17 cells leads to a condition of inflammation.

In this context, one should emphasize that obesity leads to an alteration of intestinal microbiota with an increase of Firmicutes [35], thus provoking a further aggravation of inflamm-ageing.

## Nutraceutical interventions in elderly

Nowadays, an arsenal of dietary products is available for the restoration of microbiota in young and elderly population [36]. Prebiotics, as non digestible components of fruits, vegetables and grain, are oligosaccharides able to accelerate the growth of gut anaerobes with production of SCFA [29,37]. Probiotics are viable bacteria [38] which enhance intestinal epithelial functions such as production of mucus, defensins and sIgA [39]. Moreover, probiotics upregulate phagocytic and natural killer (NK) cell functions, also inducing activation of Treg cells [40-42]. Probiotics and symbiotics (a mix of prebiotics and probiotics) have been proven to be beneficial when administered to aged people. For instance, supplementation of *Bifidobacterium (B.) lactis* HN 019 to aged individuals led to the recovery of granulocyte and NK cell activities [43]. Oral intake of *Lactobacillus (L.) pentosus* strain b240 (b240) has been shown to augment sIgA secretion in elderly people. Moreover, b240 was able to reduce frequency of common cold in aged individuals, likely acting *via* mucosal immunity [44]. In a double-blind trial *B. lactis* BL-01 and *B. bifidum* BB-02 along with inulin as a prebiotic could increase numbers of *B. bifidum* and total bifidobacteria and lactobacilli in the microbiota of elderly subjects [45]. Modification of microbiota seems to represent an essential event for less frequency of winter infections to occur. In a recent trial, administration for one month of fermented cow milk containing *L. rhamnosus* and oligofructose (a symbiotic) to free-living elderly increased serum levels of IL-1, IL-6, and IL-8, while reduced basal levels of IL-12, IL-10 and tumor necrosis factor (TNF)-α were not modified by this treatment [46]. It is likely that induction of a more vigorous acute phase response in these subjects may compensate the impaired adaptive immune response in the case of pathogen invasion.

**Figure 4:**
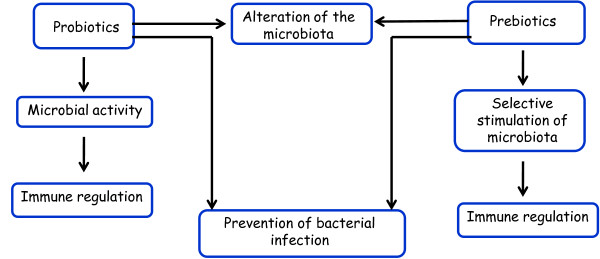
Illustration of major activities of probiotics and prebiotics.

Main functions of prebiotics and probiotics are represented in Figure 4.

Polyphenols, compounds widely present in the vegetal kingdom, have been shown to influence the composition of the gut microbiota. Consumption of blueberry [47], grape juice [48] and red wine or gin [49], respectively, mainly increased *Bifidobacterium* spp. in fecal samples from human volunteers. In addition, our recent studies have demonstrated that polyphenols contained in red wine or in fermented grape marc exhibit an anti-inflammatory role both *in vitro*[50] and *in vivo*[51]. Particularly, *in vitro* induction of human Treg cells and *in vitro* attenuation of colitis in mice with decrease of IL-1β and TNF-α content in homogenized colon seem to sustain the anti-inflammatory activities of polyphenols. Therefore, intake of dietary polyphenols in the elderly may beneficially act either on microbiota restoration and, consequentially, on attenuation of chronic inflammatory conditions.

In this framework, deficiencies of micronutrients (*e.g.,* zinc) as well as vitamin B12 have been reported in the elderly, thus accounting for frailty in the host [52,53]. However, the relationship between oligoelements and vitamin B12 and intestinal microbiota deserves further investigation in elderly.

## Conclusion

In conclusion, more studies are needed for a better comprehension of the interplay between human microbiota and gut immune cells in elderly. In fact, inter individual variations of microbiota composition mostly depending on the type of diet, life style as well use of different molecular techniques of bacterial identification seem to represent the major difficulties in this area of research. In this direction, in a very recent editorial Sartor [54] has pointed out the emergence of certain strains of sulphate-reducing Deltaprotobacteria, *e.g., Bilophila (B.) wadsworthia*, which induces colitis in mice through release of interferon-γ by Th1 cells. Quite interestingly, *B. wadsworthia* is increased in patients with ulcerative colitis, thus suggesting the need to identify new subsets of patients with IBD using Deltaprotobacteria as biomarkers [55]. It appears that consumption of saturated milk fat led to expansion of *B. wadsworthia* in mice [55]. Therefore, the possibility that also in humans changes of microbiota could be induced by milk-fat intake should be taken into consideration. On the other hand, in spite of many advances in the field of mucosal immunity, age-related changes, which occur at mucosal surface, are still not completely explored. Most of the present knowledge is related to studies in rodent models, while a few investigations have been conducted on the human aged mucosal immunity. In order to overcome this problem the use of humanized mice may help in the understanding of mucosal immunity in elderly and, for instance, constructing effective vaccines to combat infectious diseases, as well as targeting specific components of the intestinal microbiota with the supplementation of nutraceuticals seem to represent the major therapeutic intervention [56,57].

## Abbreviations

APCs: Antigen presenting cells; b240: *Lactobacillus pentosus* strain b240; CRC: Colon rectal cancer; DCs: Dendritic cells; GALT: Gut associated lymphoid tissue; IECs: Intestinal epithelial cells; IL: Interleukin; IBD: Inflammatory bowel disease; LP: Lamina propria; M: Microfolding cell; NK: Natural killer; RA: Retinoic acid; SCFA: Short chain fatty acids; SFB: Segmented filamentous bacteria; sIg: Secretory immunoglobulin; TGF: Transforming growth factor; Th: T helper; TLR: Toll-like receptor; Treg: T regulatory; TNF: Tumor necrosis factor.

## Competing interests

Both authors declare that they have no competing interests.

## Authors’ contributions

TM and EJ equally contributed. Both authors read and approved the final manuscript.

## References

[B1] Moore WE Holdeman LV Human fecal flora: the normal flora of 20 Japanese-Hawaiians Appl Microbiol 1974 27 961 979 4598229 10.1128/am.27.5.961-979.1974 PMC380185

[B2] Ley RE Peterson DA Gordon JI Ecological and evolutionary forces shaping microbial diversity in the human intestine Cell 2006 124 837 848 10.1016/j.cell.2006.02.017 16497592

[B3] Ouwehand A Vesterlund S Health aspects of probiotics IDrugs 2003 6 573 580 12811680

[B4] Zoetendal EG von Wright A Vilpponen-Salmela T Ben-Amor K Akkermans AD de Vos WM Mucosa-associated bacteria in the human gastrointestinal tract are uniformly distributed along the colon and differ from the community recovered from feces Appl Environ Microbiol 2002 68 3401 3407 10.1128/AEM.68.7.3401-3407.2002 12089021 PMC126800

[B5] Eckburg PB Bik EM Bernstein CN Purdom E Dethlefsen L Sargent M Gill SR Nelson KE Relman DA Diversity of the human intestinal microbial flora Science 2005 308 1635 1638 10.1126/science.1110591 15831718 PMC1395357

[B6] Louis P Scott KP Duncan SH Flint HJ Understanding the effects of diet on bacterial metabolism in the large intestine J Appl Microbiol 2007 102 1197 1208 10.1111/j.1365-2672.2007.03322.x 17448155

[B7] Miron N Cristea V Enterocytes: active cells in tolerance to food and microbial antigens in the gut Clin Exp Immunol 2012 167 405 412 10.1111/j.1365-2249.2011.04523.x 22288583 PMC3374272

[B8] Kraehenbuhl JP Neutra MR Epithelial M cells: differentiation and function Annu Rev Cell Dev Biol 2000 16 301 332 10.1146/annurev.cellbio.16.1.301 11031239

[B9] Iwasaki A Mucosal dendritic cells Annu Rev Immunol 2007 25 381 418 10.1146/annurev.immunol.25.022106.141634 17378762

[B10] Kanai T Mikami Y Sujino T Hisamatsu T Hibi T RORγt-dependent IL-17A-producing cells in the pathogenesis of intestinal inflammation Mucosal Immunol 2012 5 240 247 10.1038/mi.2012.6 22354322

[B11] Maldonado RA von Andrian UH How tolerogenic dendritic cells induce regulatory T cells Adv Immunol 2010 108 111 165 21056730 10.1016/B978-0-12-380995-7.00004-5 PMC3050492

[B12] Hadis U Wahl B Schulz O Hardtke-Wolenski M Schippers A Wagner N Müller W Sparwasser T Förster R Pabst O Intestinal tolerance requires gut homing and expansion of FoxP3+ regulatory T cells in the lamina propria Immunity 2011 34 237 246 10.1016/j.immuni.2011.01.016 21333554

[B13] Agace WW Persson EK How vitamin A metabolizing dendritic cells are generated in the gut mucosa Trends Immunol 2012 33 42 48 10.1016/j.it.2011.10.001 22079120

[B14] Dicarlo AL Fuldner R Kaminski J Hodes R Aging in the context of immunological architecture, function and disease outcomes Trends Immunol 2009 30 293 294 10.1016/j.it.2009.05.003 19541534

[B15] Biagi E Candela M Turroni S Garagnani P Franceschi C Brigidi P Ageing and gut microbes: perspectives for health maintenance and longevity Pharmacol Res 2012 10.1016/j.phrs.2012.10.005 23079287

[B16] Larbi A Franceschi C Mazzatti D Solana R Wikby A Pawelec G Aging of the immune system as a prognostic factor for human longevity Physiology (Bethesda) 2008 23 64 74 10.1152/physiol.00040.2007 18400689

[B17] Magrone T Jirillo E The interplay between the gut immune system and microbiota in health and disease: nutraceutical intervention for restoring intestinal homeostasis Curr Pharm Des 2013 9 7 1329 1342 23151182 10.2174/138161213804805793

[B18] Round JL Mazmanian SK Inducible Foxp3+ regulatory T-cell development by a commensal bacterium of the intestinal microbiota Proc Natl Acad Sci U S A 2010 107 12204 12209 10.1073/pnas.0909122107 20566854 PMC2901479

[B19] Davies JM Sheil B Shanahan F Bacterial signalling overrides cytokine signalling and modifies dendritic cell differentiation Immunology 2009 128 Suppl 1 e805 e815 19740342 10.1111/j.1365-2567.2009.03086.x PMC2753930

[B20] Ivanov II Atarashi K Manel N Brodie EL Shima T Karaoz U Wei D Goldfarb KC Santee CA Lynch SV Tanoue T Imaoka A Itoh K Takeda K Umesaki Y Honda K Littman DR Induction of intestinal Th17 cells by segmented filamentous bacteria Cell 2009 139 485 498 10.1016/j.cell.2009.09.033 19836068 PMC2796826

[B21] Feng T Wang L Schoeb TR Elson CO Cong Y Microbiota innate stimulation is a prerequisite for T cell spontaneous proliferation and induction of experimental colitis J Exp Med 2010 207 1321 1332 10.1084/jem.20092253 20498021 PMC2882839

[B22] Claesson MJ Cusack S O'Sullivan O Greene-Diniz R de Weerd H Flannery E Marchesi JR Falush D Dinan T Fitzgerald G Stanton C van Sinderen D O'Connor M Harnedy N O'Connor K Henry C O'Mahony D Fitzgerald AP Shanahan F Twomey C Hill C Ross RP O'Toole PW Composition, variability, and temporal stability of the intestinal microbiota of the elderly Proc Natl Acad Sci U S A 2011 108 Suppl 1 4586 4591 20571116 10.1073/pnas.1000097107 PMC3063589

[B23] Hopkins MJ Sharp R Macfarlane GT Age and disease related changes in intestinal bacterial populations assessed by cell culture, 16S rRNA abundance, and community cellular fatty acid profiles Gut 2001 48 198 205 10.1136/gut.48.2.198 11156640 PMC1728209

[B24] He T Harmsen HJ Raangs GC Welling GW Composition of faecal microbiota of elderly people Microb Ecol Health Dis 2003 15 153 159 10.1080/08910600310020505

[B25] Harmsen HJ Wildeboer-Veloo AC Grijpstra J Knol J Degener JE Welling GW Development of 16S rRNA-based probes for the Coriobacterium group and the Atopobium cluster and their application for enumeration of Coriobacteriaceae in human feces from volunteers of different age groups Appl Environ Microbiol 2000 66 4523 4527 10.1128/AEM.66.10.4523-4527.2000 11010909 PMC92335

[B26] Tiihonen K Ouwehand AC Rautonen N Human intestinal microbiota and healthy ageing Ageing Res Rev 2010 9 107 116 10.1016/j.arr.2009.10.004 19874918

[B27] Biagi E Nylund L Candela M Ostan R Bucci L Pini E Nikkïla J Monti D Satokari R Franceschi C Brigidi P De Vos W Through ageing, and beyond: gut microbiota and inflammatory status in seniors and centenarians PLoS One 2010 5 e10667 10.1371/journal.pone.0010667 20498852 PMC2871786

[B28] Pédron T Sansonetti P Commensals, bacterial pathogens and intestinal inflammation: an intriguing ménage à trois Cell Host Microbe 2008 3 344 347 10.1016/j.chom.2008.05.010 18541210

[B29] De Vuyst L Leroy F Cross-feeding between bifidobacteria and butyrate-producing colon bacteria explains bifdobacterial competitiveness, butyrate production, and gas production Int J Food Microbiol 2011 149 73 80 10.1016/j.ijfoodmicro.2011.03.003 21450362

[B30] Ioannidis O Varnalidis I Paraskevas G Botsios D Nutritional modulation of the inflammatory bowel response Digestion 2011 84 89 101 10.1159/000323456 21494040

[B31] Schiffrin EJ Morley JE Donnet-Hughes A Guigoz Y The inflammatory status of the elderly: the intestinal contribution Mutat Res 2010 690 50 56 10.1016/j.mrfmmm.2009.07.011 19666034

[B32] Manichanh C Borruel N Casellas F Guarner F The gut microbiota in IBD Nat Rev Gastroenterol Hepatol 2012 9 599 608 10.1038/nrgastro.2012.152 22907164

[B33] Kraus S Arber N Inflammation and colorectal cancer Curr Opin Pharmacol 2009 9 405 410 10.1016/j.coph.2009.06.006 19589728

[B34] Kinross J Nicholson JK Gut microbiota: dietary and social modulation of gut microbiota in the elderly Nat Rev Gastroenterol Hepatol 2012 9 563 564 10.1038/nrgastro.2012.169 22945446

[B35] Ley RE Turnbaugh PJ Klein S Gordon JI Microbial ecology: Human gut microbes associated with obesity Nature 2006 444 1022 1023 10.1038/4441022a 17183309

[B36] Candore G Caruso C Jirillo E Magrone T Vasto S Low grade inflammation as a common pathogenetic denominator in age-related diseases: novel drug targets for anti-ageing strategies and successful ageing achievement Curr Pharm Des 2010 16 584 596 10.2174/138161210790883868 20388068

[B37] Roberfroid M Gibson GR Hoyles L McCartney AL Rastall R Rowland I Wolvers D Watzl B Szajewska H Stahl B Guarner F Respondek F Whelan K Coxam V Davicco MJ Léotoing L Wittrant Y Delzenne NM Cani PD Neyrinck AM Meheust A Prebiotic effects: metabolic and health benefits Br J Nutr 2010 104 Suppl 2 S1 S63 20920376 10.1017/S0007114510003363

[B38] Hume ME Historic perspective: prebiotics, probiotics, and other alternatives to antibiotics Poult Sci 2011 90 2663 2669 10.3382/ps.2010-01030 22010256

[B39] Wallace TC Guarner F Madsen K Cabana MD Gibson G Hentges E Sanders ME Human gut microbiota and its relationship to health and disease Nutr Rev 2011 69 392 403 10.1111/j.1753-4887.2011.00402.x 21729093

[B40] de LeBlanc AM Castillo NA Perdigon G Anti-infective mechanisms induced by a probiotic Lactobacillus strain against Salmonella enterica serovar Typhimurium infection Int J Food Microbiol 2010 138 223 231 10.1016/j.ijfoodmicro.2010.01.020 20193971

[B41] Macpherson AJ Slack E The functional interactions of commensal bacteria with intestinal secretory IgA Curr Opin Gastroenterol 2007 23 673 678 10.1097/MOG.0b013e3282f0d012 17906446

[B42] Kwon HK Lee CG So JS Chae CS Hwang JS Sahoo A Nam JH Rhee JH Hwang KC Im SH Generation of regulatory dendritic cells and CD4 + Foxp3+ T cells by probiotics administration suppresses immune disorders Proc Natl Acad Sci U S A 2010 107 2159 2164 10.1073/pnas.0904055107 20080669 PMC2836639

[B43] Gill HS Rutherfurd KJ Cross ML Gopal PK Enhancement of immunity in the elderly by dietary supplementation with the probiotic Bifidobacterium lactis HN019 Am J Clin Nutr 2001 74 833 839 11722966 10.1093/ajcn/74.6.833

[B44] Shinkai S Toba M Saito T Sato I Tsubouchi M Taira K Kakumoto K Inamatsu T Yoshida H Fujiwara Y Fukaya T Matsumoto T Tateda K Yamaguchi K Kohda N Kohno S Immunoprotective effects of oral intake of heat-killed Lactobacillus pentosus strain b240 in elderly adults: a randomised, double-blind, placebo-controlled trial Br J Nutr 2012 1 10 http://dx.doi.org/10.1017/S0007114512003753 10.1017/S0007114512003753 22947249

[B45] Bartosch S Woodmansey EJ Paterson JC McMurdo ME Macfarlane GT Microbiological effects of consuming a synbiotic containing Bifidobacterium bifidum, Bifidobacterium lactis, and oligofructose in elderly persons, determined by real-time polymerase chain reaction and counting of viable bacteria Clin Infect Dis 2005 40 28 37 10.1086/426027 15614689

[B46] Amati L Marzulli G Martulli M Pugliese V Caruso C Candore G Vasto S Jirillo E Administration of a synbiotic to free-living elderly and evaluation of serum cytokines. A pilot study Curr Pharm Des 2010 16 854 858 10.2174/138161210790883633 20388097

[B47] Vendrame S Guglielmetti S Riso P Arioli S Klimis-Zacas D Porrini M Six-week consumption of a wild blueberry powder drink increases bifidobacteria in the human gut J Agric Food Chem 2011 59 12815 12820 10.1021/jf2028686 22060186

[B48] Jacobs DM Deltimple N van Velzen E van Dorsten FA Bingham M Vaughan EE van Duynhoven J (1)H NMR metabolite profiling of feces as a tool to assess the impact of nutrition on the human microbiome NMR Biomed 2008 21 615 626 10.1002/nbm.1233 18085514

[B49] Queipo-Ortuño MI Boto-Ordóñez M Murri M Gomez-Zumaquero JM Clemente-Postigo M Estruch R Cardona Diaz F Andrés-Lacueva C Tinahones FJ Influence of red wine polyphenols and ethanol on the gut microbiota ecology and biochemical biomarkers Am J Clin Nutr 2012 95 1323 1334 10.3945/ajcn.111.027847 22552027

[B50] Magrone T Marzulli G Jirillo E Immunopathogenesis of neurodegenerative diseases: current therapeutic models of neuroprotection with special reference to natural products Curr Pharm Des 2012 18 34 42 10.2174/138161212798919057 22211682

[B51] Kawaguchi K Matsumoto T Kumazawa Y Effects of antioxidant polyphenols on TNF-alpha-related diseases Curr Top Med Chem 2011 11 1767 1779 10.2174/156802611796235152 21506932

[B52] Mocchegiani E Costarelli L Giacconi R Piacenza F Basso A Malavolta M Micronutrient (Zn, Cu, Fe)-gene interactions in ageing and inflammatory age-related diseases: implications for treatments Ageing Res Rev 2012 11 297 319 10.1016/j.arr.2012.01.004 22322094

[B53] Dhonukshe-Rutten RA Lips M de Jong N Chin A Paw MJ Hiddink GJ van Dusseldorp M De Groot LC van Staveren WA Vitamin B-12 status is associated with bone mineral content and bone mineral density in frail elderly women but not in men J Nutr 2003 133 801 807 12612156 10.1093/jn/133.3.801

[B54] Sartor RB Gut microbiota: Diet promotes dysbiosis and colitis in susceptible hosts Nat Rev Gastroenterol Hepatol 2012 9 561 562 10.1038/nrgastro.2012.157 22890110

[B55] Devkota S Wang Y Musch MW Leone V Fehlner-Peach H Nadimpalli A Antonopoulos DA Jabri B Chang EB Dietary-fat-induced taurocholic acid promotes pathobiont expansion and colitis in Il10-/- mice Nature 2012 487 104 108 22722865 10.1038/nature11225 PMC3393783

[B56] Fujihashi K Kiyono H Mucosal immunosenescence: new developments and vaccines to control infectious diseases Trends Immunol 2009 30 334 343 10.1016/j.it.2009.04.004 19540811

[B57] Rehman T Role of the gut microbiota in age-related chronic inflammation Endocr Metab Immune Disord Drug Targets 2012 12 4 361 367 10.2174/187153012803832620 23017185

